# The Influence of Physical, Mental, and Cognitive Factors on Health-Related Quality of Life among Community-Dwelling Older Adults: A Focus on Central Sensitization-Related Symptoms

**DOI:** 10.3390/geriatrics9010011

**Published:** 2024-01-11

**Authors:** Yuki Kikuchi, Hideki Nakano, Akio Goda, Kohei Mori, Teppei Abiko, Nozomi Mitsumaru, Shin Murata

**Affiliations:** 1 Department of Physical Therapy, Faculty of Health Sciences, Kyoto Tachibana University, Kyoto 607-8175, Japan; nakano-h@tachibana-u.ac.jp (H.N.); abiko@tachibana-u.ac.jp (T.A.); muratas3944@gmail.com (S.M.); 2 Graduate School of Health Sciences, Kyoto Tachibana University, Kyoto 607-8175, Japan; morikouhei1@gmail.com; 3 Department of Physical Therapy, Faculty of Health and Medical Science, Hokuriku University, Ishikawa 920-1180, Japan; akio.goda@gmail.com; 4 Faculty of Allied Health Sciences, Kansai University of Welfare Sciences, Osaka 582-0026, Japan; 5 Kusukinomori Co., Ltd., Saga 848-0027, Japan; mitsumaru@kisikaisei.jp

**Keywords:** health-related quality of life, pain intensity, central sensitization-related symptoms, community-dwelling older adults

## Abstract

Most older adults wish to maintain independence in their familiar communities. However, many experience pain and pain-related disabilities which reduce their health-related quality of life (HRQOL), leading to increased hospitalizations and mortality. This study aimed to determine the impact of physical, mental, and cognitive factors, particularly central sensitization-related symptoms (CSS), on the HRQOL of community-dwelling older adults. A total of 206 participants were included in the analysis, which measured HRQOL, basic attributes, physical functions and body pain, mental factors, cognitive factors, and CSS severity using validated tools. A correlation analysis was used to examine the association between HRQOL and each measure. Furthermore, multiple regression analysis (forced entry method) was performed to identify the factors influencing the HRQOL. The study found that pain intensity and CSS severity significantly influenced the HRQOL among community-dwelling older adults. The higher the pain intensity and CSS severity, the lower their HRQOL. The participants had mild pain and CSS, demonstrating the need to monitor, address, and treat even non-severe issues in community-dwelling older adults. This association, revealed for the first time in this study, suggests that approaches to reduce pain and CSS are important for maintaining and improving the HRQOL of community-dwelling older adults.

## 1. Introduction

In 2022, the percentage of the world population aged 65 years and older was reported to be 9.7% [1]. This percentage is expected to only increase in the future, suggesting that population aging is a global concern [1]. Most older adults wish to maintain their independence in their daily lives and continue to live in familiar neighborhoods for as long as possible [2]. However, over 50% of community-dwelling older adults experience pain and pain-related disabilities [3]. Pain in older adults is associated with physical, mental, and cognitive impairments [4,5,6] and a decline in their health-related quality of life (HRQOL) [7]. Reduced HRQOL is associated with limited activities of daily living and increased frequency of hospitalization, which may reduce healthy life expectancy and increase mortality [8].

Additionally, older adults have been reported to have increased central sensitization (CS), which is associated with chronic pain [9]. It is thought to be a common pathological basis for a variety of central sensitization-related symptoms (CSS) [10], including hyperalgesia [11], fatigue [12], sleep disturbance [13], and cognitive dysfunction [14,15]. CSS are characterized by pain and complex physical and mental symptoms mainly due to central nervous system (CNS) hypersensitivity, which negatively influences patients’ HRQOL [16]. As CSS include symptoms other than pain, they may negatively affect the HRQOL of community-dwelling older adults with or without pain. Therefore, it is important to determine the impact of CSS on the HRQOL of community-dwelling older adults.

Previous studies have reported that the HRQOL decreases with more severe CSS in patients with musculoskeletal pain [17,18] and postoperative breast cancer [19]. Contrastingly, no studies have investigated the association between the HRQOL and CSS in community-dwelling older adults. Although Haruyama et al. [20] reported an association between CSS, lifestyle, and psychological factors, the HRQOL was not included as an outcome, and the impact of CSS on the HRQOL among community-dwelling older adults has not yet been established.

Therefore, this study aimed to determine the impact of physical, mental, and cognitive factors, as well as CSS severity, on the HRQOL among community-dwelling older adults. This study’s findings will contribute to the development of an approach to sustain and improve the HRQOL in community-dwelling older adults.

## 2. Materials and Methods

### 2.1. Participants

**Figure 1:**
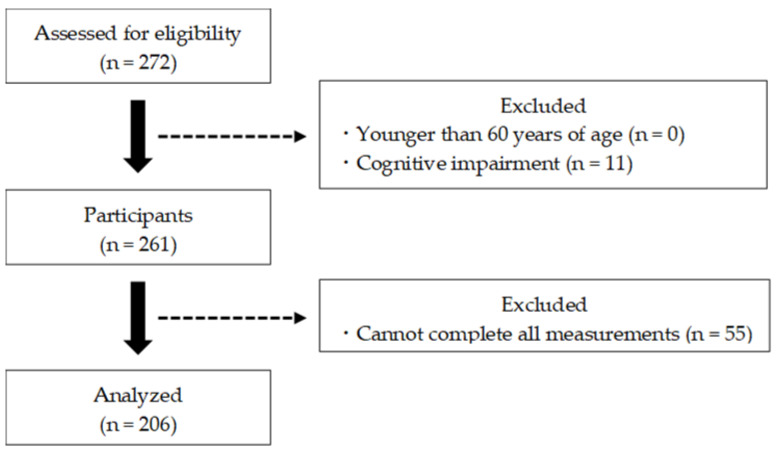
Participant selection flowchart.

For this study, 272 participants from the 2020 and 2021 health surveys conducted in Saga, Japan were screened. Data were collected on participants’ gender, age, height, weight, body mass index, and educational history. Subsequently, the participants completed physical, mental, and cognitive assessments, and the CSS severity was measured. The exclusion criteria included those younger than 60 years of age, suspected of dementia (mini-mental state examination (MMSE) score ≤23), or unable to complete all measurements. Ultimately, statistical analysis for this study was performed on 206 participants (Figure 1). The participants were informed that the data and personal information obtained through the survey would not be used for any purpose other than this research, and consent was obtained for the purpose and content of the study. This study was approved by the Research Ethics Committee of Kyoto Tachibana University (approval number: 18–26) and conducted in accordance with the Declaration of Helsinki.

### 2.2. Measures

#### 2.2.1. HRQOL

**Figure 2:**
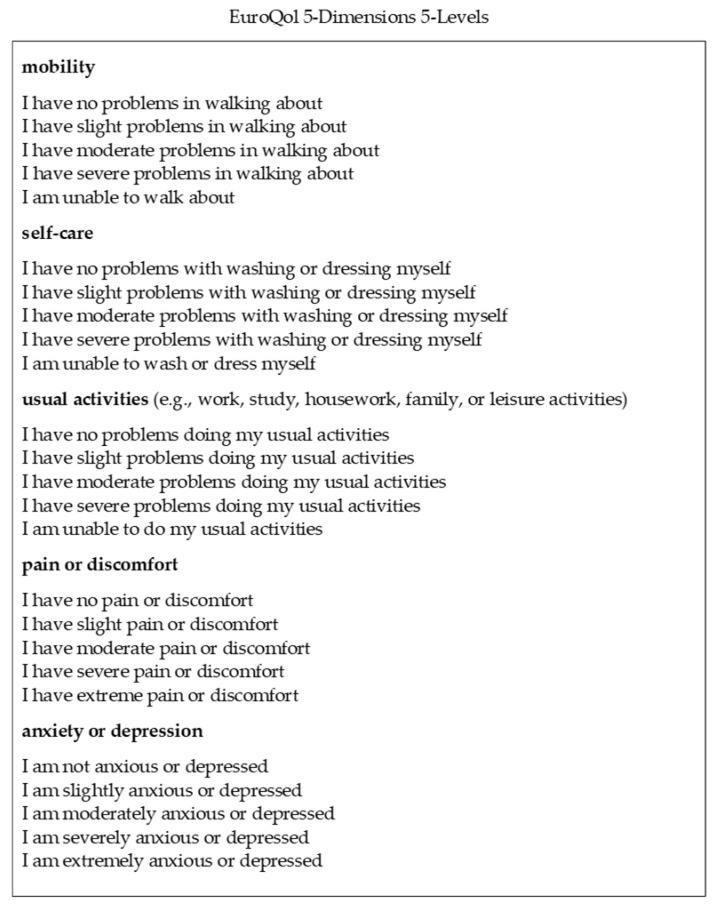
EuroQol 5 Dimensions 5 Levels system, where 1 = no problems, 2 = slight problems, 3 = moderate problems, 4 = severe problems, and 5 = extreme problems.

The HRQOL was measured using the EuroQol 5 Dimensions 5 Levels system, the details of which are shown in Figure 2 [21]. This is a self-administered questionnaire encompassing five items (mobility, self-care, usual activities, pain or discomfort, and anxiety or depression) rated on a 5 point scale (1 = no problems, 2 = slight problems, 3 = moderate problems, 4 = severe problems, and 5 = extreme problems) [22]. The five numerical combinations represent the health status of the participants, with “11111” indicating no problems at all and “55555” indicating extreme problems [23]. The combination of these values was converted into a utility value (HRQOL score), where 0 indicated death and 1 indicated perfect health, using a conversion table from the EuroQol group [23].

#### 2.2.2. Physical Factors

Physical factors were assessed with respect to physical function and pain. Grip strength and knee extension muscle strength were measured using methods described in previous studies [24]. Grip strength was measured using a digital grip strength meter (T.K.K. 5401; Takei Kiki Kogyo Co.,Niigata, Japan). At the beginning of the measurements, the limb position of the participant was in an upright posture with both upper limbs drooping and the lower limbs kept shoulder width apart. The grip strength meter was set such that the proximal interphalangeal joint angle of the finger was approximately at a 90° flexed position with the side on which the measured value was displayed. The participants gripped their hands with maximum force and were careful not to let the grip strength gauge touch their bodies or clothing. Measurements were taken twice on each side, and the average of the maximum values on the left and right sides was considered the representative value. The muscle force during knee extension was measured using a muscle force measuring table for a single leg (T.K.K. 5715, Takei Kiki Kogyo Co., Niigata, Japan). The limb of the participant at the start of the measurement was kept in a sitting position in a chair with a knee joint angle of approximately 90° in a flexed position, and a belt connected to a tension meter (T.K.K. 5710 (e), Takei Kiki Kogyo Co., Niigata, Japan) was attached to the ankle joint. The participants were instructed to extend the knee joint with maximum force. Measurements were performed twice on each side, and the mean of the maximum values on the left and right sides was considered a representative value.

To measure lower limb function, the 30-second chair stand test (CS-30) was used. The CS-30 was administered using the method described in the study by Nakatani et al. [25]. For these measurements, the examinees sat on a chair without armrests and crossed their arms in front of their chests. The examinees were given three verbal instructions: “keep arms crossed in front of the chest during the measurement”, “stand up while fully extending the knees”, and “repeat the exercise as fast as possible” [26]. The number of repetitions that could be completed within 30 s was recorded for sitting, standing, and sitting in one cycle. Cycles that were not completed within 30 s were not counted as repetitions.

The one-leg standing time was used to measure the balance capacity. The one-leg standing time was measured with reference to the open-eye one-leg standing time measurement method of Goda et al. [26]. The participants were instructed to keep both their upper limbs drooped during the measurement and to gaze at a landmark provided at eye level 2 m in front of them. Measurements ended when the raised foot touched the supporting foot or floor or when the position of the supporting foot shifted. Measurements were performed twice on each side, and the mean of the left and right measurements was considered a representative value.

The timed up and go (TUG) test was used to measure the mobility of the participants. The results were measured using the method described by Kurosawa et al. [27]. The time required to stand up from a sitting position, move around a cone 3 m away, and sit back in a chair was also measured. To ensure consistency in the results, the participants were instructed to walk “as fast as possible” during the measurement.

Pain was assessed in terms of the presence or absence of pain, pain intensity, and the number of pain sites. Participants responded to the question “Have you had any physical pain in the last month” with “yes” or “no” [28]. Furthermore, the number of pain sites selected from the options “head”, “neck”, “shoulder”, “back”, “hip”, “knee joint”, and “ankle joint” was counted in response to the question “Where in the body do you have pain” [28]. The pain intensity was measured using a numerical rating scale that has shown reliability and validity [29]. Participants responded on an 11 point scale ranging from “not painful at all” (0 points) to “exclusively painful” (10 points).

#### 2.2.3. Mental Factors

The mental factors for depressive tendencies were assessed using the Geriatric Depression Scale (GDS), a depression scale developed for older adults by Yesavage [30]. For each item, the response options were “yes” or “no”. A negative response received a score of 1, and a positive response received a score of 0, with higher scores indicating greater depressive tendencies [30]. Our study used the GDS-5 [31,32], which has been shown to be a reliable and valid shortened version of the GDS.

#### 2.2.4. Cognitive Factors

In this study, cognitive and attentional functions were assessed as cognitive factors. Cognitive function was assessed using the MMSE developed by Folstein et al. [33] to assess general cognitive function. The MMSE is internationally popular as a screening test for dementia and is characterized by task contents like writing, sentence construction, and graphical imitation. The maximum score is 30 points, and a person is suspected to have dementia if they obtained a score of 23 points or less [34].

Attentional functioning was assessed using the Trail Making Test (TMT). The TMT part A (TMT-A) [35,36], which has been shown to be reliable and valid, was administered in this study. The participants were presented with a piece of paper with random numbers from 1 to 25 and were asked to connect the numbers with a line in ascending order. The time taken to complete the task (connecting all numbers) was measured.

#### 2.2.5. Central Sensitization-Related Symptoms (CSS)

**Figure 3:**
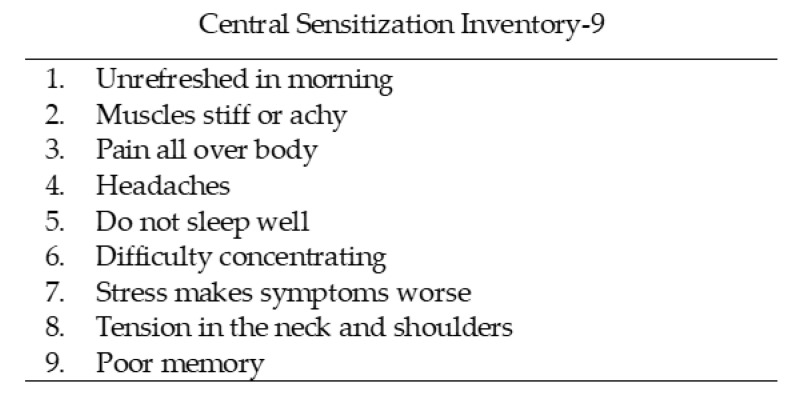
Short form of the Central Sensitization Inventory, where 0 = never, 1 = rarely, 2 = sometimes, 3 = often, and 4 = always.

CS is defined as increased sensitivity of the nociceptive neurons in the CNS to normal or subthreshold ascending stimuli [37]. It is a common pathological basis of CSS which cause a range of physical and psychological symptoms [38]. CSS severity is commonly assessed using the Central Sensitization Inventory (CSI) [39]. The original CSI consists of Part A, with 25 questions on CSS, and Part B, which asks whether there is a diagnosis for 8 CSS [40]. In this study, the CSS severity was assessed using a shortened version of the CSI, namely the CSI-9 [40]. The CSI-9 has been shown to be highly reliable and valid as a screening tool for CSS in clinical practice. The items for the CSI-9 question-and-response options are shown in Figure 3 [40]. The participants were asked to answer each question on a 5 point scale: 0 = never, 1 = rarely, 2 = sometimes, 3 = often, and 4 = always (Figure 3). The CSI-9 scores range from 0 to 36, with higher scores indicating greater CSS severity [40]. In previous studies, a CSI-9 score ≥20 has been defined as the cut-off value for determining severe CSS [41].

### 2.3. Statistical Analysis

Analyses were performed on 206 participants after excluding those who met the exclusion criteria. Correlation analyses were performed between the HRQOL and physical, mental, and cognitive factors as well as CSS severity. Moreover, for independent variables that were significantly correlated with the HRQOL, multiple regression analysis (forced entry method) was performed with the HRQOL as the dependent variable to investigate the factors influencing it. Multiple regression analysis considered multicollinearity by calculating the variance inflation factor (VIF) and checking whether the VIF was <5 [42]. Statistical analysis was performed using IBM SPSS Statistics for Windows, Version 29.0 (Armonk, NY, USA), with a significance level of 5%.

## 3. Results

Table 1 shows the basic attributes; HRQOL; physical, mental, and cognitive factors; and CSS severity of the 206 participants. Regarding the basic attributes, the participants’ mean age was 77.4 (6.0) years, the mean height was 151.8 (11.6) cm, the mean weight was 51.9 (8.9) kg, the mean body mass index was 22.6 (3.4) kg/m^2^, and the mean educational history was 11.6 (2.3) years. The mean (standard deviation) HRQOL score for the main outcome was 0.896 (0.120). The mean (standard deviation) CSI-9 score was 6.5 (4.8). Five (2.4%) participants had a score above the cut-off value of 20, which was used to determine severe CSS.

Correlation analysis revealed significant positive correlations between the HRQOL and knee extension muscle strength (r = 0.156, *p* = 0.025), CS-30 score (r = 0.267, *p* < 0.001), one-leg standing time (r = 0.189, *p* = 0.006), and MMSE score (r = 0.172, *p* = 0.013). Furthermore, there was a significant positive correlation between the HRQOL and age (r = −0.157, *p* = 0.024), while the TUG test score (r = 0.259, *p* < 0.001), GDS-5 score (r = −0.268, *p* < 0.001), pain intensity (r = −0.456, *p* < 0.001), number of pain sites (r = −0.418, *p* < 0.001), and CSI-9 score (r = −0.409, *p* < 0.001) showed a significant negative correlation (Table 2 and Table 3).

Furthermore, multiple regression analysis was performed with the HRQOL as the dependent variable and items that showed significant correlations with the HRQOL (age, knee extension, CS-30 score, one-leg standing time, TUG test score, pain intensity, number of pain sites, GDS-5 score, MMSE score, and CSI-9 score) as the independent variables. The analysis identified pain intensity (β = −0.217, *p* = 0.017) and CSI-9 score (β = −0.277, *p* < 0.001) as factors significantly influencing the HRQOL in community-dwelling older adults (Table 4). The VIF between the independent variables ranged from 1.093 to 2.344, and there were no concerns about multicollinearity.

## 4. Discussion

The mean HRQOL score was 0.896 ± 0.120. Shiroiwa et al. [43] studied HRQOL in a randomly selected sample of Japanese community-dwelling older adults in their 70s and reported scores of 0.889 ± 0.154 for men and 0.876 ± 0.157 for women. The participants in this study had HRQOL scores that were approximately those of general community-dwelling older adults despite being older. The mean ± standard deviation of the CSI-9, a measure of CSS severity, was 6.5 ± 4.8 points, and five participants (2.4%) exceeded the cut-off value of 20 points [42]. In a study by Haruyama et al. [20], 4.2% of community-dwelling older adults in Japan reported severe CSS, and the participants in this study had similar values. Therefore, our participants were similar to community-dwelling older adults in previous studies with regard to general characteristics.

This study investigated the impact of physical, mental, and cognitive factors and CSS severity on the HRQOL of community-dwelling older adults. The results showed that pain intensity and CSS severity were factors influencing the HRQOL among community-dwelling older adults. Our findings suggest the importance of approaching not only pain but also CSS severity to maintain and improve the HRQOL among community-dwelling older adults. The association between HRQOL and CSS severity in community-dwelling older adults has not been previously reported and was revealed for the first time in this study.

CSS is a collective term for the range of symptoms that occur with CS as a common pathological basis. These include pain, fatigue, sleep disturbances, and cognitive dysfunction. CSS other than pain are often considered to be complaints and are neglected [16]. However, these symptoms are also associated with decreased HRQOL and have been reported to be a factor in increasing the risk of future chronic pain [16,44,45,46,47]. Moreover, physical symptoms that cannot be explained by organic factors reported in the general population may be differentiated by the CSI, enabling a more specific approach [48,49]. Thus, it is critical for healthcare workers to understand pain and non-painful CSS conditions and work to maintain and improve HRQOL in community-dwelling older adults through appropriate coping to reduce symptoms. Previous studies have shown that manual and exercise therapy are effective in improving CSS and pain [50,51]. Improvements in symptoms with exercise therapy have also been reported for pain, chronic fatigue syndrome [52], sleep disorders [53], and cognitive dysfunction [54]. This suggests that an approach centered on exercise therapy has the potential to improve CSS and maintain or improve the HRQOL in community-dwelling older adults. Aerobic exercise is known to reduce pain sensitization by activating descending pain suppression mechanisms and endogenous opioid and cannabinoid systems [51]. However, there is no settled view on the effective intensity, duration, and frequency of prescribing aerobic exercise, which needs to be further investigated through intervention studies.

The association between pain and HRQOL has been investigated in several previous studies [55,56,57]. In this study, both pain intensity and the number of pain sites were significantly correlated with the HRQOL, whereas only pain intensity was selected as the factor significantly influencing the HRQOL. These results support the study by Cedraschi et al. [55], which found that pain intensity, rather than the number of pain sites, suggests a more important association with the HRQOL among community-dwelling older adults. Furthermore, coping with pain becomes more difficult with age in older people, owing to reduced physical, sensory, and other functions [58]. This may result in increased disability and an impact on the quality of life. The mean pain intensity of the participants was mild, being 2.4 points, but it was an important factor affecting the HRQOL. Our results suggest that even mild pain may reduce the HRQOL of community-dwelling older adults. Consequently, careful monitoring, coping, and treatment of pain is critical to the HRQOL of community-dwelling older adults.

This is the first study to determine the impact of pain intensity and CSS severity on the HRQOL in community-dwelling older adults. Koga et al. [16] reported that the higher the pain intensity, the more severe the CSS. This suggests that pain intensity and CSS may form a vicious cycle and negatively affect the HRQOL. To maintain and improve the HRQOL in community-dwelling older adults, symptom reduction should be addressed by considering both pain intensity and CSS. Hence, to maintain and improve the HRQOL of community-dwelling older adults, attention should be paid not only to pain intensity but also to CSS.

However, because this was a cross-sectional study, it was not possible to determine a causal relationship between the pain intensity, CSS, and HRQOL. Further longitudinal studies are required to elucidate the causal relationship between pain intensity, CSS, and HRQOL and to devise effective approaches for community-dwelling older adults.

## 5. Conclusions

This study revealed that not only pain intensity but also CSS status affects the HRQOL in community-dwelling older adults. This study is the first to show that CSS influences the HRQOL in community-dwelling older adults. Pain intensity and CSS may interact with each other, forming a vicious cycle. The findings of this study therefore suggest that both pain intensity and CSS status need to be monitored for coping and treatment to maintain and improve the HRQOL in community-dwelling older adults. In addition, future research could clarify the causal relationship between HRQOL, pain intensity, and CSS severity in community-dwelling older adults, which could lead to more effective interventions and contribute to improving their HRQOL.

## Figures and Tables

**Table 1:** Characteristics of participants.

n = 206			
			Mean ± SD
Age	years		77.4 ± 6.0
Gender	n (%)	Male/Female	38 (18.4)/168 (81.6)
Height	cm		151.8 ± 11.6
Weight	kg		51.9 ± 8.9
BMI			22.6 ± 3.4
Educational history	years		11.6 ± 2.3
HRQOL score			0.896 ± 0.120
Hand grip	kgf		21.8 ± 6.1
Knee extension	kgf		24.2 ± 7.7
CS-30	repetitions		20.1 ± 6.2
One-leg standing time	seconds		31.6 ± 35.9
TUG	seconds		7.0 ± 3.9
Pain	n (%)		119 (57.8)
Pain intensity	points		2.3 ± 2.4
Number of pain sites	n (%)	0	87 (42.2)
		1	60 (29.1)
		2	42 (20.4)
		3	14 (6.8)
		4	2 (1.0)
		5	-
		6	1 (0.5)
GDS-5	points		0.7 ± 1.0
MMSE	points		28.2 ± 1.9
TMT	seconds		137.7 ± 53.3
CSI-9	points		6.5 ± 4.8

Abbreviations : BMI = body mass index, HRQOL = health-related quality of life, CS-30 = 30-second chair stand test, TUG = timed up and go test, GDS-5 = Geriatric Depression Scale-5, MMSE = mini-mental state examination, TMT = trail making test, CSI-9 = Central Sensitization Inventory-9, and SD = standard deviation.

**Table 2:** Correlations of HRQOL score with basic attributes.

n = 206					
	Age	Height	Weight	BMI	Educational History
HRQOL score	−0.157 *	−0.048	−0.031	−0.013	0.048

Abbreviations: HRQOL score = health-related quality of life score and BMI = body mass index. * *p* < 0.05.

**Table 3:** Correlations of HRQOL score with physical, mental, and cognitive factors and CSS.

n = 206						
	**Hand Grip**	**Knee Extension**	**CS-30**	**One-Leg Standing**	**TUG**	
HRQOL score	0.114	0.156 *	0.267 **	0.189 **	−0.259 **	
	**Pain intensity**	**Number of pain sites**	**GDS-5**	**MMSE**	**TMT-A**	**CSI-9**
HRQOL score	−0.456 **	−0.418 **	−0.268 **	0.172 *	−0.069	−0.409 **

Abbreviations : HRQOL score = health-related quality of life score, CSS: central sensitization-related symptoms, CS-30: 30-second chair stand test, TUG = timed up and go test, GDS-5 = Geriatric Depression Scale-5, MMSE = mini-mental state examination, TMT = trail making test, and CSI-9: Central Sensitization Inventory-9. * *p* < 0.05. ** *p* < 0.01.

**Table 4:** Multiple regression analysis results.

n = 206							
Dependent Variables	HRQOL Score						
		B	β	95% CI		*p* Value	VIF
				Lower	Upper		
Independent variables	Age	−0.002	−0.085	−0.004	0.001	0.219	1.381
	Knee extension	0.000 ^a^	−0.032	−0.003	0.002	0.635	1.320
	CS-30	0.002	0.081	−0.001	0.004	0.275	1.563
	One-leg standing time	0.000 ^b^	0.011	0.000	0.000	0.873	1.353
	TUG	0.002	0.058	−0.002	0.005	0.352	1.093
	Pain intensity	−0.011	−0.217	−0.020	−0.002	0.017	2.344
	Number of pain sites	−0.014	−0.126	−0.034	0.006	0.161	2.315
	GDS-5	−0.004	−0.029	−0.019	0.012	0.657	1.253
	MMSE	0.006	0.101	−0.002	0.015	0.116	1.191
	CSI-9	−0.007	−0.277	−0.010	−0.003	*p* < 0.001	1.474
	Adjusted R^2^	0.287					

Abbreviations : 95% CI = 95% confidence interval, VIF = variance inflation factor, HRQOL score = health-related quality of life score, CS-30 = 30-second chair stand test, TUG = timed up and go test, GDS-5 = Geriatric Depression Scale-5, MMSE = mini-mental state examination, CSI-9 = Central Sensitization Inventory-9. ^a^ = − 0.000496939026249198; ^b^ = 0.0000365406170752391.

## Data Availability

The data presented in this study are available upon request from the corresponding author. The data are not publicly available because they contain information that may infringe on the privacy of the study participants.
